# Supportive Management of Patients with Advanced Pheochromocytomas and Paragangliomas Receiving PRRT

**DOI:** 10.3390/curroncol28040247

**Published:** 2021-07-26

**Authors:** Erica S. Tsang, Gayle Funk, Janet Leung, Grace Kalish, Hagen F. Kennecke

**Affiliations:** 1 Division of Medical Oncology, BC Cancer, Vancouver, BC V5Z 4E6, Canada; erica.tsang@bccancer.bc.ca; 2 Virginia Mason Cancer Institute, Seattle, WA 98101, USA; gayle.funk@virginiamason.org (G.F.); Janet.Leung@virginiamason.org (J.L.); Grace.Kalish@virginiamason.org (G.K.); 3 Providence Cancer Institute & Chiles Research Institute, Portland, OR 97213, USA

**Keywords:** pheochromocytoma, paraganglioma, peptide receptor radionuclide therapy

## Abstract

Peptide receptor radionuclide therapy (PRRT) is used to treat patients with advanced malignant pheochromocytomas (PCCs) and paragangliomas (PGLs). Patients are at risk of a PRRT-induced catecholamine crisis, and standard guidelines regarding the prevention and management of infusion reactions are lacking. In this case series, the institutional experience of five sequential patients with metastatic PCCs and PGLs receiving PRRT on an outpatient basis is described, of which four had symptomatic tumors and three had a high burden of disease. All patients with symptomatic tumors were treated with preventive management prior to the initiation of PRRT, and no infusion reactions or catecholamine crises were documented. PRRT may be delivered safely on an outpatient basis for patients with metastatic PCCs and PGLs with the involvement of an interdisciplinary team.

## 1. Introduction

Pheochromocytomas (PCCs) and paragangliomas (PGLs) represent rare neuroendocrine tumors with variable 5-year survival outcomes in the metastatic setting, ranging from 34–69% [1]. There are limited treatment options for metastatic PCCs and PGLs, and current options include surgical tumor debulking, external beam radiotherapy, cytotoxic chemotherapy, and targeted agents such as sunitinib [2,3,4,5,6,7]. In 2018, peptide receptor radionuclide therapy (PRRT) was approved in the United States for the treatment of advanced gastroenteropancreatic neuroendocrine tumors (GEP-NETs) with positive expression of somatostatin receptors [8]. PRRT has also been approved in Canada for patients with unresectable midgut NETs after progression on a somatostatin analogue. Similar to GEP-NETs, the majority of PCCs and PGLs are also somatostatin-receptor positive, providing the biological rationale for PRRT [9]. Specifically, the components of PRRT, including the radionuclide Lutetium-177, peptide, and chelator DOTA, bind to somatostatin receptors on the PCC or PGL tumor cell membrane. This subsequently leads to internalization and local delivery of beta-radiation from Lutetium-177, causing cell damage and death [10]. While there are no prospective phase III data documenting the efficacy of PRRT in PCCs and PGLs, the current literature consists of retrospective studies documenting that PRRT improves outcomes for these patients [11,12,13,14]. The recently published North American Neuroendocrine Tumor Society (NANETS) consensus guidelines for PCCs and PGLs suggest participation in a clinical trial if PRRT is being considered [15].

Hormonal crises of PCCs and PGLs precipitated by PRRT are well documented and are generally described in the initial 24–48 h of the first cycle of PRRT as a result of excessive catecholamine secretion [16]. In one of the largest published series of PRRT-induced hormonal crisis, the overall rate of reactions was 6/479 (1%) patients, of which 1/3 (33%) of patients with PCCs developed a hormonal crisis [16]. In another report, 2/3 (66%) of PGL patients treated with PRRT developed catecholamine crisis [17]. Among patients with advanced PGL/PCC treated with iobenguane I 131, a total of 11% of 68 patients developed increased blood pressure, all within 24 h of treatment [18]. PRRT protocol modifications, including lengthening infusion time and decreasing upfront treatment dosage, have been suggested to mitigate or prevent adverse reactions, such as a catecholamine crisis or tumor lysis syndrome [17]. There are currently no clear guidelines or recommendations regarding supportive or preventive management of infusion reactions with PRRT [2,19].

In this article, the single institutional experience of five sequential patients with metastatic PCCs and PGLs initiated on PRRT in the outpatient setting between 2018 and 2020 is described. Preventive and symptom management during the course of therapy are described (Table 1) and treatment outcomes are summarized.

## 2. Case 1

**Figure 1:**
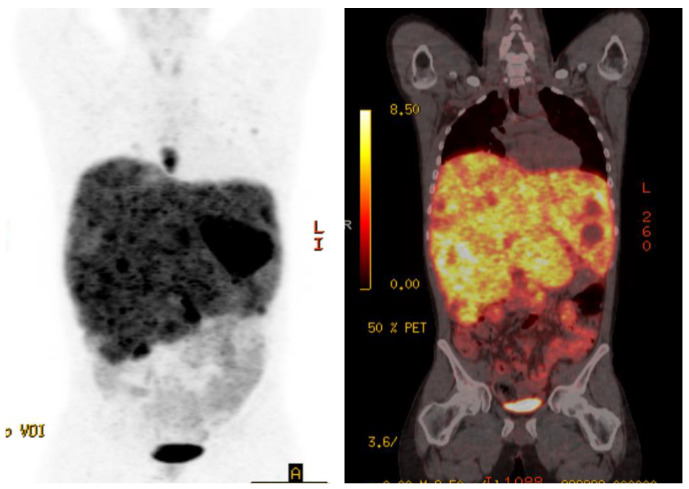
Case 1, Baseline Gallium-68 Dotatate PET-CT showed avid lesions in the lungs, posterior mediastinum, liver, retroperitoneal adenopathy, and diffuse liver and lung metastases.

Case 1 is a 28-year-old male initially diagnosed at the age of 24 with abdominal pain in the context of refractory hypertension. MIBG scan demonstrated avidity in an adrenal mass and liver lesions. He underwent a left adrenalectomy, with pathology confirming a 6.5 cm pheochromocytoma, Ki-67 not reported. Germline testing revealed no germline abnormalities. Due to extensive disease burden, the liver lesions were unresectable and the patient sought alternative treatments for three years until he re-presented to medical attention with significant weight loss, fatigue, and protuberant hepatomegaly. Restaging scans showed significant progression including a left paraspinal mass, retroperitoneal adenopathy, and diffuse liver and lung metastases. Gallium-68 Dotatate PET-CT scan demonstrated avid lesions in the lungs, posterior mediastinum, liver, retroperitoneal lymph nodes, and several bones in the axial skeleton (Figure 1). Twenty-four-hour epinephrine measured 558 mcg (normal range 0.5–20 mcg/24 h) and norepinephrine 2959 mcg (normal range 15–80 mcg/24 h). Serum free metanephrines measured 130 nmol/L (normal range <0.5 nmol/L) and normetanephrine >273 nmol/L (normal range <0.9 nmol/L). Liver enzymes were elevated at ALT 79 IU/L (normal range 29–33) and AST 73 IU/L (normal range 5–40). Due to the high burden of disease, alpha-, beta- and calcium channel blockers were initiated for blood pressure and symptom control prior to initiation of PRRT to reduce the risk of a symptomatic crisis related to the infusion. Therapy was commenced with doxazosin 8 mg twice daily, nifedipine ER 30 mg three times daily, and propranolol 120 mg ER once daily.

A total of two cycles of full dose Lutetium-177 Dotatate, 7.4 GBq (200 mCi) given every 8 weeks, were administered without complications or hemodynamic instability. Due to uncontrolled hypertension at baseline that was unresponsive to oral alpha-, beta- and calcium channel blocker therapy, intravenous (IV) calcium channel blocker therapy was commenced 60 min prior to cycle 1 to titrate blood pressure, and PRRT was given at the standard rate and dose. At the time of cycle 2 of PRRT, blood pressure was well controlled with oral therapy, and symptoms of catecholamine excess had significantly improved, in addition to overall well-being, and the patient gained back a significant amount of weight. Cycle 2 was administered at standard dose and rate, without complications or use of IV anti-hypertensives. Unfortunately, the control of symptoms was not durable and within 3 months of commencing PRRT, the symptoms of pain, hypertension, fatigue and weight loss recurred and the patient chose not to continue PRRT and no alternate therapy was given.

## 3. Case 2

Case 2 is a 28-year-old male who initially presented at the age of 25 with right flank pain and underwent a right adrenalectomy and nephrectomy, with pathology consistent with pheochromocytoma. Genetic testing revealed no germline abnormalities. Postoperative MIBG and catecholamines were negative. Two years later, he developed pelvic pain in the right lower quadrant and unintendedly lost 40 kg of weight, subsequently presenting with an ECOG performance status of 3. He endorsed occasional palpitations, shortness of breath on exertion, and bone pain. Gallium 68-Dotatate PET-CT demonstrated extensive thoracic and abdominal nodal uptake, bone uptake, with T11 vertebral and right anterior iliac crest involvement and liver metastases. Symptomatic vertebral bony metastases were treated with external beam radiation therapy, which was completed six weeks prior to the start of PRRT. Treatment with prazosin 2 mg once daily and metoprolol ER 25 mg twice daily was initiated prior to cycle 1 of PRRT. Full dose Lutetium-177 Dotatate, 7.4 GBq (200 mCi), was administered at standard rate without any hemodynamic instability or symptoms.

At the time of the second cycle of PRRT, a significant improvement in symptoms was observed, ECOG improved to 1 and he had regained 10 kg. Prior to his fourth cycle of PRRT, palpitations and hypertension had resolved and alpha- and beta-blocker therapy could be discontinued. Four cycles of PRRT therapy were administered and a near complete radiographic response was achieved in sites of nodal and liver metastasis. A total of 10 months after starting PRRT, symptoms of hypertension, pain and weight loss recurred and the patient was diagnosed with progressive liver, nodal and bone metastases.

## 4. Case 3

**Figure 2:**
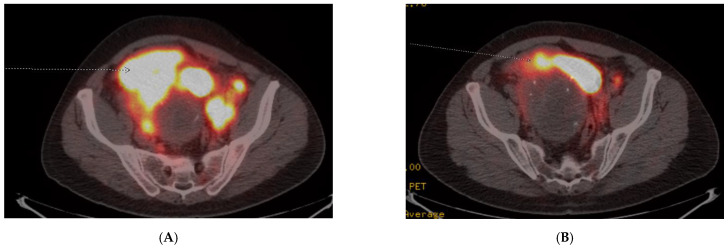
Case 3, (**A**) Baseline Gallium-68 Dotatate PET-CT demonstrated a large pelvic mass with retroperitoneal adenopathy and ipsilateral ureteric compression; (**B**) Follow-up Gallium-68 Dotatate PET-CT after 3 cycles of Lu-177 Dotatate demonstrated significant reduction in pelvic mass and retroperitoneal adenopathy.

A 45-year-old previously healthy male presented with episodic fevers and lower back pain, along with 10 kg intentional weight loss. Initial CT scan showed a large pelvic mass with retroperitoneal adenopathy and ipsilateral ureteric compression. Biopsy of the pelvic mass demonstrated a paraganglioma, with a Ki-67 of 30–40%. Given the rapid progression of disease, he received six cycles of carboplatin and etoposide first-line for six cycles. His pelvic pain, fevers and night sweats improved after the third cycle but recurred within one month of discontinuing chemotherapy. A Gallium-68 PET-CT was performed to determine eligibility for PRRT and documented heterogeneous uptake of a large dominant pelvic mass and extensive retroperitoneal adenopathy, Krenning score 4 (Figure 2A). The remainder of his imaging revealed extensive new onset right sided hydronephrosis and left supraclavicular adenopathy. The patient initiated second-line therapy with PRRT, prior to which he was started on prazosin 2 mg twice daily and atenolol 25 mg once daily. Symptoms significantly improved by the second cycle of PRRT and a total of four planned treatments of Lutetium-177 Dotatate, 7.4 GBq (200 mCi), were given without adverse reaction. A radiographic partial response to therapy was documented on follow-up Gallium-68 Dotate PET-CT (Figure 2B) and he discontinued both alpha and beta-blocker therapy until 8 months after PRRT treatment began, when his symptoms recurred and he was diagnosed with progressive retroperitoneal metastasis.

## 5. Case 4

A 52-year-old female underwent a left adrenalectomy and nephrectomy for a pheochromocytoma at age 32. Twenty-four-hour urine metanephrines were negative at the time. At age 50, she had presented with lower back pain, sciatic numbness and paresthesias. MRI spine demonstrated a large mass involving L3–L5, and she underwent decortication surgery, with improvement in her neurological symptoms, but significant residual disease remained which was unresectable. FDG PET-CT revealed a distant thoracic lymph node and Gallium-68 Dotatate PET/CT documented somatostatin receptor avid disease, Krenning score 4, in the surgical tumor bed at L3–L5 and the thoracic lymph node. Twenty-four-hour urine metanephrines and normetanephrines were elevated at 31 mcg and 1020 mcg, respectively. Genetic testing revealed a germline *SDHB* mutation (c.423 + 1G > A). Prior to initiating PRRT, she was started on doxazosin 2 mg once daily and atenolol 12.5 mg once daily.

After cycle 1 of Lutetium-177 Dotatate, 7.4 GBq (200 mCi), she developed significant fatigue and was found to have low blood pressure at 90/50. After holding atenolol, her symptoms resolved and she continued on doxazosin alone until her fourth cycle, after which it was discontinued due to the absence of symptoms. Follow-up Gallium-68 PET/CT documented diminished somatostatin receptor expression in both areas of disease, and she remained progression free 5 months after starting PRRT. After completing four cycles of PRRT, she was subsequently lost to follow-up.

## 6. Case 5

**Figure 3:**
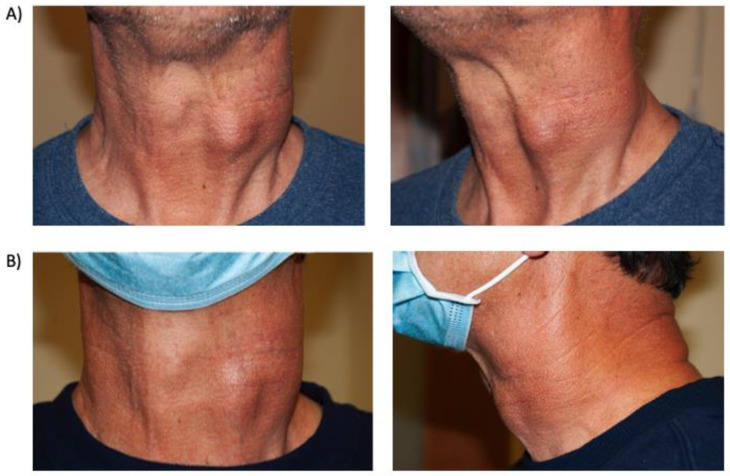
Case 5, (**A**) Pre-treatment anterior and lateral images of left carotid body paraganglioma; (**B**) Post-treatment anterior and lateral images of left carotid body paraganglioma for Case 5.

A 61-year-old man who since the age of 16 had noted a very gradually progressive left sided neck mass was eventually diagnosed at the age of 29 with a left carotid body paraganglioma, which was only partially resected due to invasion of the carotid artery. By the age of 45, the tumor measured 10 cm and CT imaging documented a mass encompassing the common carotid to above the level of the bifurcation without airway impingement. Surveillance continued without surgery, radiation or systemic therapy, and an eventual diagnosis of a germline *SDHB* mutation was made. By age 60, the mass became more symptomatic and an additional nodule developed anterolaterally, associated with regional discomfort and dysphagia, but no hypertension, tachycardia or other systemic symptoms. Surgery was considered but deferred by the patient due to the need for extensive vascular reconstruction and morbidity. FDG/PET-CT documented intense uptake of the tumor surrounding the carotid artery and surrounding level IIA/B nodes but not distant metastasis. Gallium-68 PET-CT documented intense Krenning grade 4 positive tumor and lymph nodes and treatment was commenced with Lutetium-177 Dotatate. Due to the absence of symptoms including hypertension and tachycardia and no measurable increase in serum catecholamines and metanephrines except a mild elevation in plasma dopamine, no alpha or beta blockades were instituted prior to PRRT. A total of four cycles of PRRT therapy were tolerated with exception of fatigue during the first 2 weeks of therapy and the mass palpably reduced in size and became more mobile. Serial photographs documented a significant reduction in tumor size (Figure 3).

## 7. Discussion

In this article, a modern case series of five patients with advanced, unresectable PCCs and PGLs who received Lutetium-177 Dotatate therapy is presented. Four of the five patients experienced a durable clinical and radiographic benefit, with often a dramatic clinical response early on after receiving PRRT. Outcomes are consistent with other retrospective case series, which include partial responses in 2 of 28 patients (7%) and stable disease in 13 of 28 patients (46%) reported in one cohort [14,20]. A prospective single-arm clinical trial of Lutetium-177 Dotatate in patients with inoperable PCCs and PGLs is currently being conducted (NCT03206060), and we anticipate that this will provide further data with regard to efficacy.

The duration of response ranged from 3 to 10 months among three patients. The fourth patient was lost to follow-up, and the fifth patient recently completed four cycles of PRRT and continues with ongoing follow-up at the time of manuscript submission. Previous retrospective case studies have reported a mean duration of response of approximately 18 months, with median progression-free survival reported between 17 and 39 months [11,13,20]. It is challenging to compare treatment duration directly given the limited sample size and significant disease burden in our case series.

No infusion reactions or catecholamine crises were observed with any of the five patients, four of whom had symptomatic tumors and three of whom had very bulky disease. The lack of infusion reactions in this patient series may be related to the routine use of pre-medications administered to all symptomatic patients prior to the first cycle of PRRT. This approach included a combined alpha- and beta-adrenergic blockade and is similar to the perioperative management of PCCs and PGLs for surgical resection. We have outlined our recommendations for pre-PRRT management using combined alpha- and beta-adrenergic blockade in Table 2, with the aim to maintain blood pressure at ≤120/80 mmHg and heart rate <100 beats per minute (bpm). In the case of the second patient (Case 2), due to the presence of uncontrolled hypertension in spite of maximal oral alpha-, beta and calcium channel blockade, the decision was made to deliver the first initial of PRRT in a monitored setting to allow for the titration of IV calcium channel blocker therapy, which was not required for subsequent cycles. This highlights that appropriate pre-medication with combined alpha and beta blockade can allow for safe and effective outpatient delivery of PRRT.

Taken together with the current evidence in the literature for the safety and efficacy of PRRT in this setting, PRRT can be delivered safely on an outpatient basis with the involvement of an interdisciplinary team.

## Figures and Tables

**Table 1:** Characteristics of five patients diagnosed with pheochromocytoma/paraganglioma treated with PRRT.

Case	Diagnosis	Sites of Metastasis	Functional Symptoms	Day 1, Cycle 1 Symptom Management Medications *	PRRT Infusion Reactions
Case 1	28 y.o. male, left adrenal pheo age 24, recurred age 25.	Bone, nodal, >75% liver, lung	Severe weight loss, uncontrolled HTN, tachy.	Doxazosin 8 mg po bid. Nifedipine 30 mg ER po tid. Propranolol 120 mg ER po qd	No reactions NOTE: Due to uncontrolled baseline hypertension, IV calcium channel blocker therapy was given during cycle
Case 2	28 y.o. male, right adrenal pheo age 25, recurred age 27.	Bone, nodal, liver, lung	Severe weight loss, HTN, tachy, constipation, anxiety, palpitations.	Prazosin 2 mg qd (held after cycle 3) Metoprolol 25 mg extended release BID (held after cycle 3)	None
Case 3	45 y.o. male, right pelvic mass and adenopathy, para on biopsy.	Soft tissue pelvic mass, hydronephrosis and nodes	Severe weight loss, tachy, night sweats, HTN.	Prazosin 2 mg PO BID Atenolol 25 mg PO once daily (held after cycle 2)	None
Case 4	52 y.o. female, SDHB mut, age 21 left adrenal pheo, relapsed age 51 with spinal metastases.	L3–L5, thoracic node	Tachy	Doxazosin 2 mg PO BID (held after cycle 4) Atenolol 12.5 mg PO once daily (held after cycle 1)	None
Case 5	61 y.o. male, germline SDHB mut, carotid body para age stable over 31 years, transformed to rapidly progressive at age 61.	Carotid artery invasion and regional nodes	None	None	None

Abbreviations: y.o.: years old; Pheo: pheochromocytoma; Para: paraganglioma; HTN: hypertension; tachy: tachycardia; mut: mutation. * Pre-medications were started several weeks prior to scheduled PRRT treatment, with target blood pressure <120/80 mmHg.

**Table 2:** Recommended approach of combined alpha- and beta-adrenergic blockade for PCCs and PGLs to maintain blood pressure at ≤120/80 mmHg and heart rate <100 bpm prior to initiating PRRT.

Blockade Strategy	Examples of Medications Used
(1) Alpha-adrenergic blockade	- Selective alpha-1-adrenergic blockers: prazosin, terazosin, doxazosin - Phenoxybenzamine is often used preoperatively, but is not preferred for longer term use
(2) Beta-adrenergic blockade	- Metoprolol, propranolol, atenolol *
(3) Calcium channel blockade	- Amlodipine, nicardipine This class of drugs is often used to augment blood pressure control with combined alpha- and beta-adrenergic blockade

NOTE: Metyrosine is another agent typically used perioperatively, but we would not recommend this given that PRRT is not limited to one cycle. Long-term use of metyrosine can result in side effects, such as sedation, anxiety, depression and extrapyramidal signs. * Beta-blockers should be started only after alpha-blockers to prevent unopposed alpha-adrenergic receptor stimulation, which could lead to increased blood pressure.

## Data Availability

The data presented in this study are available in this article.
